# A Parametric Study of Epoxy-Bonded CF/QF-BMI Composite Joints Using a Method Combining RBF Neural Networks and NSGA-II Algorithm

**DOI:** 10.3390/polym17131769

**Published:** 2025-06-26

**Authors:** Xiaobo Yang, Xingyu Zou, Jingyu Zhang, Ruiqing Guo, He Xiang, Lihua Zhan, Xintong Wu

**Affiliations:** 1 College of Mechanical and Electrical Engineering, Lanzhou Jiaotong University, Lanzhou 730070, China; lzjt_yangxb@163.com (X.Y.); 13026466860@163.com (X.Z.); 15092331955@163.com (J.Z.); 2 Aerospace Research Institute of Materials & Technology, Beijing 100079, China; 3 Ningbo Branch of Ordnance Science Institute of China, Ningbo 315103, China; xianghe68@126.com; 4 Institute of Light Alloy, Central South University, Changsha 410083, China; yjs-cast@csu.edu.cn; 5 School of Advanced Manufacturing, Nanchang University, Nanchang 330029, China

**Keywords:** carbon-fiber-reinforced bismaleimide composite, quartz-fiber-reinforced bismaleimide composite, epoxy-bonded joint, RBF neuron machine learning, NSGA-II algorithm, finite element simulation model, tensile and shear strength

## Abstract

The epoxy-bonded joint between carbon-fiber-reinforced bismaleimide (CF-BMI) and quartz-fiber-reinforced bismaleimide (QF-BMI) composites can meet the structure–function integration requirements of next-generation aviation equipment, and the structural design of their bonding zones directly affects their service performance. Hence, in this study, the carbon-fiber-reinforced bismaleimide composite ZT7H/5429, the woven quartz-fiber-reinforced bismaleimide composite QW280/5429, and epoxy adhesive film J-116 were used as research materials to investigate the influence of the bonding area size on the mechanical properties, and this study proposes a novel design methodology combining radial basis function (RBF) neuron machine learning with the NSGA-II algorithm to enhance the mechanical properties of the bonded components. First, a finite element simulation model considering 3D hashin criteria and cohesion was established, and its accuracy was verified with experiments. Second, the RBF neuron model was trained using the finite element tensile strength and shear strength data from various adhesive layer parameter combinations. Then, the multi-objective parameter optimization of the surrogate model was accomplished through the NSGA-II algorithm. The research results demonstrate a high consistency between the finite element simulation results and experimental outcomes for the epoxy-bonded CF/QF-BMI composite joint. The stress distribution of the adhesive layers is similar under the different structural parameters of adhesive films, though the varying structural dimensions of the adhesive layers lead to distinct failure modes. The trained RBF neuron model controls the prediction error within 2.21%, accurately reflecting the service performance under various adhesive layer parameters. The optimized epoxy-bonded CF/QF-BMI composite joint exhibits 16.1% and 11.2% increases in the tensile strength and shear strength, respectively.

## 1. Introduction

Carbon-fiber-reinforced bismaleimide and quartz-fiber-reinforced bismaleimide composites demonstrate application potential in the aerospace field because of their significantly low density, high specific strength, and integrated molding capabilities. CF-BMI composites enable substantial strength enhancement for components and are an ideal choice for the main load-bearing structures of fuselages in the aerospace field [1,2]; QF-BMI composites meet the stringent requirements for wave-transparent functional components because of their excellent dielectric constant [3]. The adhesive joining of CF-BMI and QF-BMI composites enables the synergistic utilization of their respective advantages, thereby satisfying the structural–functional integration requirements of next-generation aviation equipment. Currently, in the field of aerospace bonding applications, the primary adhesive types used in bonded structures include adhesive films, liquid adhesives, and powder adhesives. Compared to other adhesive types, the epoxy-based film adhesive offers distinct advantages for precision-joining multiple composite materials due to the controllable thickness, solvent-free composition, and suitable and stable mechanical properties with a superior bonding strength. At present, with the technological iteration of advanced aviation equipment for supersonic cruise capabilities and full-band electromagnetic countermeasure systems, higher requirements are placed on the manufacturing of high-quality epoxy-bonded CF/QF-BMI composite joints [4,5].

Currently, epoxy-bonded CF/QF-BMI composite joints for aviation use mostly adopt the secondary bonding process [6,7,8]. The first step is to manufacture carbon-fiber-reinforced bismaleimide composite components and quartz-fiber-reinforced bismaleimide composite components. In the next step, the bonding mechanism of the epoxy-based film adhesive encompasses both physical transformations and intricate chemical curing reactions, which collectively determine the joint performance. The phase transition from solid to liquid allows the epoxy-based film adhesive to infiltrate and dissolve the composite surface, while the concurrent epoxy-BMI copolymerization establishes an interpenetrating crosslinked interface between the CF-BMI composite component and the QF-BMI composite component. Within the optimal temperature window, the epoxy film forms superior bonding interfaces through complete crosslinking without inducing thermal degradation. And the curing pressure critically governs the bonding quality through void suppression, enhanced resin flow, and uniform adhesive distribution. Consequently, the autoclave process has become the industry-standard approach for manufacturing epoxy-bonded composite components, enabling simultaneous dimensional accuracy and mechanical property enhancement through controlled thermo-mechanical conditions. Notably, the structural characteristics of the adhesive layer zone are critical factors that directly affect the component bonding performance while ensuring process stability [9,10]. Therefore, a rational and effective dimensional design of the bonding zone proves essential for enhancing the service performance of epoxy-bonded CF/QF-BMI composite joints.

Composite research teams have investigated the impact of the dimensional design of the bonding zone on the service performance from three aspects, i.e., experimental testing, simulation modeling, and structural optimization. According to Pisharody et al., the experimental failure load of a carbon fiber reinforced plastics (CFRPs) component decreases as the adhesive layer thickness increases, and the increase in both shear stress and delamination stress in the first layer of adherents adjacent to the bonding layer leads to a decrease in bonding strength [11]. Chen et al. proposed that the shear strength of CFRP single-lap joints gradually decreases as the bonding length and the adhesive layer thickness increase [12]. Therefore, the optimal selection of the lap length and thickness of the adhesive layer significantly improves the tensile–shear strength in single-lap joints. Sun et al. investigated the tensile performance of CFRP single-lap joints through experiments and numerical simulations and employed cohesive zone models and the 3D hashin model to study the fracture mechanism for hybrid modes of bonded composites [13]. During the modeling of single-lap joints, Kim et al. fully considered the coupling failure characteristics of joint adhesive failure and the delamination and failure of the fiber and the matrix in the adhesive layer and matrix. They further worked on numerical examples, which have effectively demonstrated that the cohesion model and 3D hashin model can accurately predict the coupling characteristics of adhesive bonding failure and delamination under the maximum load [14]. Yang et al. achieved a 15.0% performance improvement in CFRP single-lap joints by using the dimensions of optimal joints of an ACO-BP neural network within the specified range [15]. Li et al. developed a novel and improved method supported by the deep neural network model and fruit fly optimization algorithm to predict the aluminum plate–CFRP plate joint failure load and effectively optimize the joint design based on the prediction results [16]. The current research primarily involves single-material composite bonded components and metal-composite bonded components [17,18,19], with a limited investigation into the adhesive layers of epoxy-bonded CF/QF-BMI composite joints and without any clarity on the failure mechanism for epoxy-bonded CF/QF-BMI composite joints. Moreover, existing studies on the bond area dimensions of the bonded joints predominantly combine experiments with simulations to determine the typical dimensions. There are few methods to establish a surrogate model by means of machine learning, and then use the multi-objective algorithm to obtain the optimal dimensions of the bonding zone.

Therefore, based on experiments performed herein on single-lap joints of CF/QF-BMI composites, a finite element model considering 3D hashin and cohesion is established in this study. Then, a design method, i.e., Latin hypercube sampling, is used to generate 300 groups of bonding dimension samples of varying thicknesses, widths, and lengths, and these 300 models are subject to an Abaqus simulation. After that, the RBF neuron model is employed to establish the surrogate model based on the finite element results. Finally, the NSGA-II algorithm is adopted to achieve the multi-objective optimization of the surrogate model, clarify the influence of the adhesive layer width, length, and thickness on the component bonding performance, and provide a novel methodology for designing the bond area dimensions of epoxy-bonded CF/QF-BMI composite joints.

## 2. Specimen Preparation and Experimental Tests

### 2.1. Material and Sample Preparation

**Figure 1:**
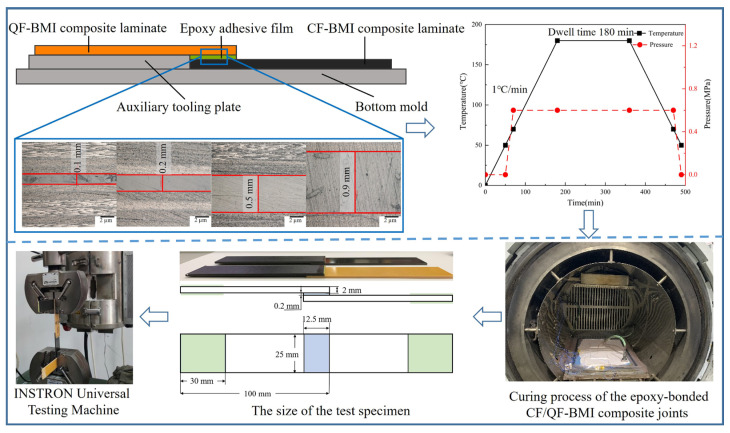
The schematic of preparation and testing specimens of epoxy-bonded CF/QF-BMI composite joints.

The existing literature indicates that the adhesive layer thickness exerts a significant impact on the performance of bonded components [7,9]. Meanwhile, this provides a verification basis for the subsequent finite element modeling. Therefore, experiments on the preparation and single lapping of epoxy-bonded CF/QF-BMI composite joints with varying adhesive layer thicknesses were carried out. Carbon-fiber-reinforced bismaleimide composite (ZT7H/5429), woven quartz-fiber-reinforced bismaleimide composite (QW280/5429), and epoxy adhesive film J-116 were used for the bonding experiments. All materials and auxiliary materials used for the experiments were procured from Kuang-Chi Technologies Co., Ltd., Shenzhen, China. The CF-BMI composite laminate had a stacking sequence of [45°/90°/–45°/0°/45°]_s_, while the QF-BMI composite laminate had a stacking sequence of [45°/90°/–45°/0°/45°/90°/–45°/0°/45°/90°]_s_. Both the CF-BMI and QF-BMI composite laminates had a thickness of 2 mm, and the J-116 adhesive film had a single-layer thickness of 0.1 mm. In the process of the experiments, the adhesive film thickness control was achieved using the rigid spacer method illustrated in Figure 1, and the thickness difference between auxiliary tooling plate and the CF-BMI composite laminate ensured that the adhesive thickness had exactly the target value. Adhesive thicknesses of 0.1 mm, 0.2 mm, 0.5 mm, and 0.9 mm were used to investigate the impact of the adhesive thickness on the performance of epoxy-bonded CF/QF-BMI composite joints. Four auxiliary tooling plates with thicknesses of 2.1 mm, 2.2 mm, 2.5 mm, and 2.9 mm are fabricated to achieve the target adhesive film thicknesses. In order to achieve high-precision adhesive thickness control, the auxiliary tooling plates must maintain a thickness tolerance below 0.01 mm, while the composite laminates require a thickness variation of less than 0.01 mm. As shown in the local metallographic images, the thickness tolerance of the adhesive films across varying dimensions was maintained below 0.02 mm. The autoclave curing process was adopted for fabricating epoxy-bonded CF/QF-BMI composite joints [20,21]. The curing process involved heating the epoxy-bonded CF/QF-BMI joints to 180 °C at 1 °C/min with a 180 min isothermal hold, following the manufacturer’s recommended curing schedule. Autoclave pressure was systematically increased to 0.6 MPa when the component temperature reached 50 °C and maintained throughout the curing cycle until process completion. The autoclave used for component bonding and molding was manufactured by the Dalian Sakurada Company.

### 2.2. Performance Test and Characterization

The single-lap experiment was used to test the mechanical properties of the epoxy-bonded CF/QF-BMI composite joints. According to the national standard GB/T-7124 [22], the size of the specimen was 100 mm × 25 mm × 2 mm, and the size of the bonded area was 12.5 mm × 25 mm. Reinforcing plates were used as the reinforcement sheets at both ends of the specimen for avoiding the damage at the ends of the test specimen caused by the fixture during the loading process. The INSTRON universal testing machine was used for the single-lap experiment. Its tensile speed was 1.5 mm/min, and its data recording rate was 1 Hz. The experiment was stopped when the adhesive layer was completely damaged, and 5 repeated tests were carried out for each group. Meanwhile, in order to study the influence of the bonding length, width, and thickness on the mechanical properties of the epoxy-bonded CF/QF-BMI composite joints, the tensile strength and lap shear strength were determined, and their expressions are as follows:

(1) $$\sigma_{T}=\frac{F_{T,max}}{W\cdot T}$$

(2) $$\sigma_{S}=\frac{F_{T,max}}{W\cdot S}$$

where $F_{T,max}$ is the ultimate tensile load (N), S is the bonding length (mm), W is the bonding width (mm), and T is the thickness of the composite laminate (mm). At the same time, a scanning electron microscope (SEM model: TESCAN MIRA3 LMU) was used to characterize the failure mode of the specimen.

## 3. Theoretical Model

### 3.1. Intrinsic Model and Failure Criteria

During modeling, both the CF-BMI and QF-BMI composite materials in a single layer are regarded as equivalent homogeneous materials with fiber orientation [23]. When the epoxy-bonded CF/QF-BMI composite joint is subjected to a tensile load, the fibers and the resin matrices of the CF-BMI composite material and the QF-BMI composite material may become damaged. Therefore, based on the 3D hashin failure criterion [24,25], this study aims to predict the four progressive failure processes of the CF-BMI and QF-BMI composite laminates: (1) fiber tensile failure; (2) fiber compression failure; (3) matrix tensile failure; and (4) matrix compression failure. The specific formulae are shown as follows:

Fiber tensile failure:
  (3) $${\left(\frac{\sigma_{11}}{X_{t}}\right)}^{2}+\frac{1}{S_{12}^{2}}{\left(\sigma_{12}^{2}+\sigma_{13}^{2}\right)}^{2}\leq 1$$
Fiber compression failure:
  (4) $${\left(\frac{\sigma_{11}}{X_{c}}\right)}^{2}\leq 1$$
Matrix tensile failure:
  (5) $$\frac{1}{Y_{t}^{2}}{\left(\sigma_{22}^{2}+\sigma_{33}^{2}\right)}^{2}+\frac{1}{S_{23}^{2}}(\sigma_{23}^{2}-\sigma_{22}\sigma_{33})+\frac{1}{S_{12}^{2}}(\sigma_{12}^{2}+\sigma_{13}^{2})\leq 1$$
Matrix compression failure:
  (6) $$\frac{1}{Y_{c}}[({\frac{Y_{c}}{2S_{23}})}^{2}-1](\sigma_{22}+\sigma_{33})+\frac{1}{{4S}_{23}^{2}}{{(\sigma }_{22}+\sigma_{33})}^{2}+(\sigma_{23}^{2}-\sigma_{22}\sigma_{33})+\frac{1}{S_{12}^{2}}(\sigma_{12}^{2}+\sigma_{13}^{2})\leq 1$$
  where $X_{t}$ and $Y_{t}$ represent the longitudinal tensile strength and the transverse tensile strength, respectively. $X_{c}$ and $Y_{c}$ represent the longitudinal compressive strength and the transverse compressive strength, respectively. Notably, the longitudinal direction is designated as the fiber direction, and the transverse direction is designated as perpendicular to the fiber direction. $\sigma_{ij}$ represents the stress components, $S_{ij}$ represents the shear strength, and the indices i and j take the values of 1, 2, or 3. Index 1 corresponds to the fiber direction, while indices 2 and 3 represent directions perpendicular to the fiber.

In this study, the bilinear cohesive model is used to predict the crack formation and evolution process in the adjacent interface layers of the composite laminate [19,26,27,28,29]. The constitutive relationship of the cohesive element in the elastic stage is as follows:

(7) $$\left[\begin{array}{c} t_{n}\\ t_{s}\\ t_{t} \end{array}\right]=\left[\begin{array}{ccc} K_{nn} & 0 & 0\\ 0 & K_{ss} & 0\\ 0 & 0 & K_{tt} \end{array}\right]\left[\begin{array}{c} \varepsilon_{n}\\ \varepsilon_{s}\\ \varepsilon_{t} \end{array}\right]$$

where $t_{n}$ and $\varepsilon_{n}$ represent the normal stress and strain, respectively. $t_{s}$ and $t_{t}$ represent the shear stress in the shear direction. $\varepsilon_{s}$ and $\varepsilon_{t}$ represent the shear strain in the shear direction. $K_{nn}$, $K_{ss}$, and $K_{tt}$ represent the stiffness.

The quadratic stress criterion is used to predict the initiation of interfacial damage, and the formula is as follows:

(8) $${\left\{\frac{\langle t_{n}\rangle }{t_{n}^{0}}\right\}}^{2}+{\left\{\frac{\langle t_{s}\rangle }{t_{s}^{0}}\right\}}^{2}+{\left\{\frac{\langle t_{t}\rangle }{t_{t}^{0}}\right\}}^{2}=1$$

where $t_{n}^{0}$ represents the interfacial tensile strength, and $t_{s}^{0}$ and $t_{t}^{0}$ represent the interfacial shear strengths. As the load increases, the crack propagates, and the interface between layers. Meanwhile, the stiffness of the cohesive element continuously decreases until its removal from the mesh.

The damage evolution of the cohesive element is governed by a power exponential criterion, and the formula is as follows:

(9) $${\left\{\frac{\langle G_{I}\rangle }{G_{IC}}\right\}}^{\lambda }+{\left\{\frac{\langle G_{II}\rangle }{G_{IIC}}\right\}}^{\lambda }+{\left\{\frac{\langle G_{III}\rangle }{G_{IIIC}}\right\}}^{\lambda }=1$$

where $G_{I}$, $G_{II},$ and $G_{III}$ represent the interfacial energies of pure types I, II, and III, respectively. $G_{IC}$, $G_{IIC},$ and $G_{IIIC}$ represent the critical energy release rates. λ represents the mixed-mode fracture correction factor.

The epoxy adhesive film is defined as isotropic, and the ductile damage criterion is adopted to determine whether it is damaged. This criterion considers that the equivalent plastic strain ${\bar{\varepsilon }}_{d}^{pl}$ at the initiation of material damage is a function of the stress triaxiality η and the strain rate ${\dot{\bar{\varepsilon }}}^{pl}$, as follows:

(10) $${\bar{\varepsilon }}_{d}^{pl}={\bar{\varepsilon }}_{d}^{pl}(\eta ,{\dot{\bar{\varepsilon }}}^{pl})$$

where the subscript d represents the damage initiation of the epoxy adhesive film, pl is an abbreviation for plasticity, η=p/q represents the triaxial stress, p represents the pressure stress, q represents the von Mises equivalent stress, and ${\dot{\bar{\varepsilon }}}^{pl}$ represents the equivalent plastic strain rate. When the determination condition for the initiation of damage is verified to be valid, the state parameter $w_{d}$ satisfies the following condition:

(11) $$w_{d}=\int \frac{d{\bar{\varepsilon }}^{pl}}{{\bar{\varepsilon }}_{d}^{pl}(\eta ,{\dot{\bar{\varepsilon }}}^{pl})}=1$$

During the analysis, the increment in ${∆w}_{d}$ for each incremental step is calculated as follows:

(12) $${∆w}_{d}=\frac{∆{\bar{\varepsilon }}^{pl}}{{\bar{\varepsilon }}_{d}^{pl}\left(\eta ,{\dot{\bar{\varepsilon }}}^{pl}\right)}\geq 0$$

After the damage is initiated, the stiffness of the epoxy adhesive film gradually decreases according to the specific damage evolution law determined based on the energy dissipated during the damage process. Meanwhile, the reduction in stiffness caused by the failure mechanism can be modeled with a scalar damage variable $s_{i}\;\left(i\in N_{act}\right)$:

(13) $$s_{i}=\frac{L{\dot{\bar{\varepsilon }}}^{pl}}{{\bar{u}}_{f}^{pl}}=\frac{{\dot{\bar{u}}}_{f}^{pl}}{{\bar{u}}_{f}^{pl}}$$

where the equivalent plastic displacement can be obtained using ${\bar{u}}_{f}^{pl}=\frac{2G_{f}}{\sigma_{y0}}$. L represents the unit feature length, f represents the final damage state of the epoxy adhesive film, and $\sigma_{y0}$ is the yield stress corresponding to the failure situation. In this study, the fracture energy ($G_{f}$) is set to 0.5 kJ/m^2^.

During the analysis, the scalar damage equation can be used to determine the stress tensor at any moment:

(14) $$\sigma =(1-D)\bar{\sigma }$$

where σ represents the undamaged stress tensor calculated in the increment step. $\bar{\sigma }$ represents the stress of the material when it is undamaged, and D represents the overall damage variable. The adhesive layer elements no longer have the bearing capacity when the overall damage variable D reaches a certain threshold value.

### 3.2. Finite Element Simulation Model

**Figure 2:**
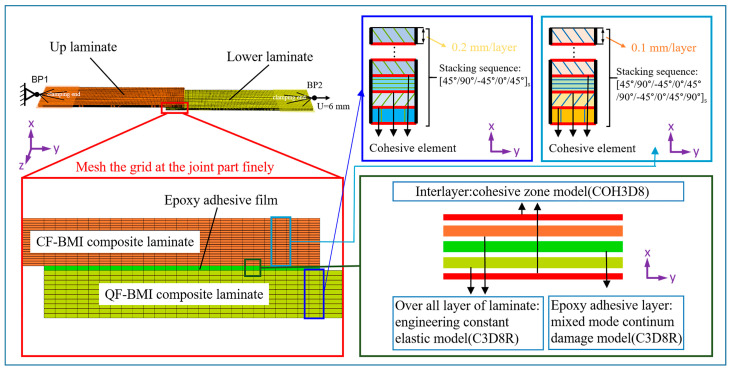
Schematic illustration of a finite element model for a single-lap joint structure made of CF/QF-BMI composite laminate.

A three-dimensional finite element model of the epoxy-bonded CF/QF-BMI composite joint is established using the commercial Abaqus software. The setup of the CF-BMI and QF-BMI composite laminates is consistent with the experimental design. At this time, it is assumed that the carbon and quartz fibers are uniformly distributed within the two types of laminates. The thickness of each layer of the CF-BMI composite laminate is 0.1 mm, and that of each layer of the QF-BMI composite laminate is 0.2 mm. As mentioned in Ref. [11], a 3D numerical modeling for the prediction of mixed failure characteristics in composite single-lap joints with a hybrid adhesive layer, the thickness of the interface between two adjacent bonded layers is assumed to be 5–10% of the prepreg thickness. Therefore, in this study, the thickness of the interface between layers is set to 0.005 mm. The thicknesses of the adhesive layers are set to 0.1 mm, 0.2 mm, 0.5 mm, and 0.9 mm, respectively. Notably, when providing data samples for machine learning, the structural dimensions of the finite element model change according to the data from the hyper-Latin sampling. During the meshing process, the mesh length in the bonded area is 0.5 mm, and the mesh length in the rest of the parts is 1 mm. The mesh width is consistent with the single-layer thickness of various materials, as shown in Figure 2.

Linear hexahedral elements such as C3D8R are used for the CF-BMI and QF-BMI composites and the epoxy adhesive film, while eight-node cohesive elements such as COH3D8 are used for the interface elements. The clamping parts at both ends of the model adopt the distributed coupling type for the point–surface coupling, which restricts the movement of the coupling surface to the translation and rotation of the reference node. A fixed constraint is applied at the coupling point of the CF-BMI composite laminate, and a displacement load in the X-direction is applied to the end of the QF-BMI composite laminate with the maximum displacement of 6 mm. The smooth step is used during the loading process. The explicit dynamics is used in the simulation solution process, and the mass is scaled by a factor of 100. The material parameters required for the CF-BMI composite, woven QF-BMI composite, and epoxy adhesive film in the simulation are shown in Table 1 and Table 2.

### 3.3. Machine-Learning Model

The surrogate model learns the input and output data of the finite sample array and uses an approximate mathematical model to analyze the input and output data for approximating the results and, then, for obtaining the approximate relationship between the inputs and outputs. However, due to the complexity, nonlinearity, and other characteristics of the surrogate model, the requirements for selecting sample points during the establishment of the surrogate model are increasingly stringent. The Latin hypercube sampling design demonstrates a superior sample point generation accuracy for nonlinear problems [33,34], thus gaining popularity in selecting machine-learning sample points. In the aviation industry, the adhesive thickness below 0.1 mm may compromise the formation of a continuous adhesive film, while the thickness exceeding 2 mm may lead to a reduction in shear stress transfer efficiency. Therefore, the adhesive thickness range is selected as [0.1, 2] mm. Meanwhile, based on the dimensional ranges documented in references [7,9,10,15], the length range of [10, 70] mm and the width range of [10, 70] mm are selected as research ranges, which can simultaneously address the computational efficiency and maintain the representativeness of the calculation results. It should be noted that the parameter ranges for the adhesive thickness, length, and width can be extended according to the actual engineering needs. Latin hypercube sampling generates 300 sample points within this range, and the tensile and shear properties of the epoxy-bonded CF/QF-BMI composite joints are obtained through a finite element simulation of these sampled dimensions.

Since the RBF neural network has a good function approximation capability and other characteristics, its learning algorithm can be trained according to the given data to approximate the complex nonlinear relationship [35,36]. Therefore, in this study, the RBF neural network surrogate model is used to establish the surrogate model for the tensile strength and shear strength of the single-lap structure of the epoxy-bonded CF/QF-BMI composite joint. To enhance the training efficiency and accuracy, data preprocessing is required. The mechanical data of the tensile strength and shear strength corresponding to the geometric parameters of different interfaces derived from the Abaqus simulation modeling are normalized to the same range [0 1]. In order to improve the training efficiency and accuracy, a data preprocessing method is required. The mapminmax function is selected [37], and its expression is as follows:

(15) $$y=(y_{max}-y_{min})\times \frac{\left(x-x_{min}\right)}{\left(x_{max}-x_{min}\right)}+y_{min}$$

where x represents the simulation data from [$x_{min}$ $x_{max}$], and the normalized data y is limited in the range of [$y_{min}$ $y_{max}$].

**Figure 3:**
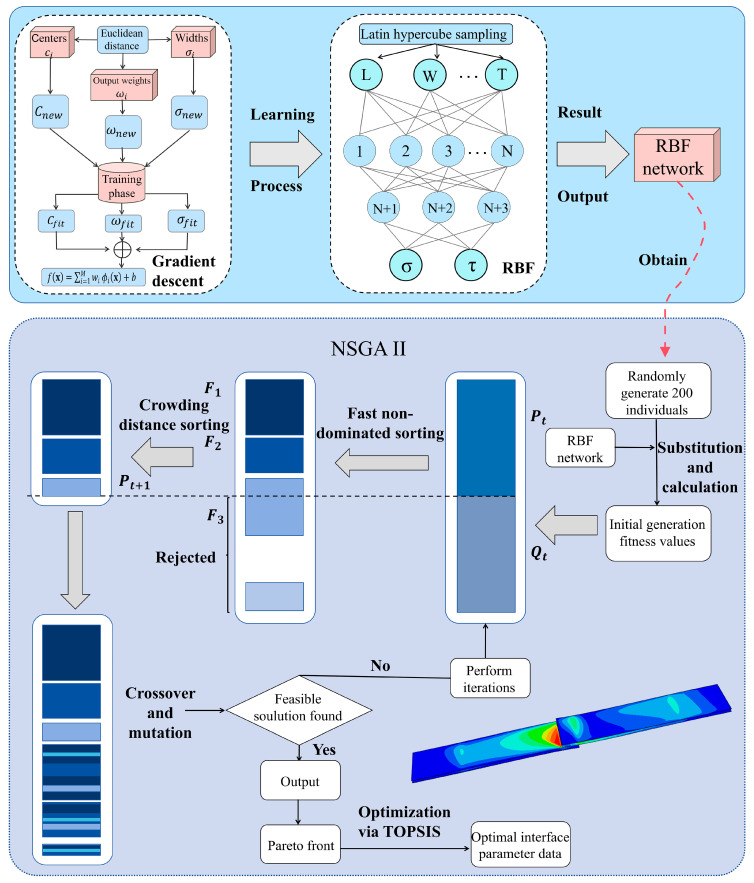
RBF neural network machine-learning fitting process schematic diagram and NSGA-II algorithm flowchart diagram.

As shown in Figure 3, the RBF neural network model adopts a three-layer feedforward network topology. The first layer plays a role in transmitting data information by inputting the length, width, and thickness parameters of joints into the model; the second layer is a hidden layer, and the number of neurons in the hidden layer is set as per the empirical Equation (16). By using Equation (17) and the power series expansion method, linearly inseparable length/width/thickness vectors are transformed into linearly separable ones. The kernel function for a hidden layer neuron is a Gaussian function (18), which is used to calculate the Euclidean distance between the input data sample value and the center point [38]. Such a distance is used as the response value of the activation function. Higher response values indicate a better parameter alignment of the neurons in the fitting layer with fitting objectives, signifying a better fitting performance. In the case of a lower response value, the gradient descent method is used for the interpolation of neuron parameters to constantly improve the response value until it meets the targets. Moreover, the third layer is the output layer, the information of the fitting neuron is transmitted to the output layer for linear fitting and weighting, and gradient descent is performed on the weighted value until the fitting model meets the target requirements, as shown in Equation (19). This process outputs a model of fitting between the geometric parameters and mechanical properties of the epoxy-bonded CF/QF-BMI composite joint:

(16) $$n_{h}=\sqrt{n_{i}+n_{o}}+N$$

where $\;n_{i}$ and $n_{o}$ denote the number of neurons in the input and output layers, respectively, and N denotes a constant whose range is [l 10]. Its exact value is determined by the complexity of the model.

(17) $$K^{\prime }(\vec{X_{i}},\vec{X_{j}})=\sum_{n=0}^{+\infty }\frac{(\vec{X_{i}}\cdot \vec{X_{j}}{)}^{n}}{\sigma^{n}n!}$$

In the formula, the vector X generates a form similar to polynomial expansion. Through parameter mapping, the data are transformed from being low-dimensional to high-dimensional:

(18) $$\psi_{m}\left(x\right)=exp\left(-\frac{∥x-c_{m}{∥}^{2}}{2\sigma_{m}^{2}}\right)$$

where x and $c_{m}$ denote the input feature vector and the center of the m-th basis function, respectively. $\sigma_{m}$ is the width of the m-th basis function, which determines the extent of the function’s spread. $∥x-c_{m}{∥}^{2}$ represents the square of the Euclidean distance, which is the distance from point x to the center point $c_{m}$:

(19) $$\theta_{j}^{k+1}=\theta_{j}^{k}-\alpha \frac{\partial J(\theta^{k})}{\partial \theta_{j}},j=0,1,\cdots ,n$$

where $\theta_{j}^{k}$ is the value of parameter $\theta_{j}$ at the k-th iteration. α is the learning rate, which serves to regulate the step size during each update. Additionally, $\frac{\partial J(\theta^{k})}{\partial \theta_{j}}$ represents the partial derivative of the objective function concerning parameter $\theta_{j}$ during the k-th iteration.

Once the surrogate model linked by tensile strength, shear strength, and the geometric parameters of the adhesive interface has been established, it is imperative that we perform an error analysis on the model to guarantee its predictive accuracy. To assess the fitting accuracy of the surrogate model, the coefficient of determination (R^2^), the mean absolute error (MAE), and the mean bias error (MBE) are introduced [39]. The value of $R^{2}$ falls within the range of 0 to 1. When the value approaches 1, it indicates that the model can better fit the data. The expression is as follows:

(20) $$R^{2}=1-\frac{\sum_{i=1}^{n}(y_{i}-\hat{y_{i}}{)}^{2}}{\sum_{i=1}^{n}(y_{i}-\bar{y}{)}^{2}}$$

The MAE is a quantitative metric, which represents the average absolute difference between values predicted by a regression model and true values. By calculating the mean of the absolute deviations, the MAE offers an intuitive measure of prediction accuracy. A smaller value of MAE indicates that predictions and true values have a closer agreement, signifying more precise model performance. The expression for the MAE is as follows:

(21) $$MAE=\frac{1}{n}\sum_{i=1}^{n}|y_{i}-{\hat{y}}_{i}|$$

The MBE is a metric that measures the average relative deviation between predicted values obtained from the regression model and observed true values. The MBE incorporates the proportion of errors relative to actual values. A smaller MBE indicates a higher prediction accuracy. The expression for the MBE is as follows:

(22) $$MBE=\frac{1}{n}\sum_{i=1}^{n}\frac{y_{i}-{\hat{y}}_{i}}{y_{i}}$$

where n denotes the number of samples, $y_{i}$ denotes the estimated value, and ${\hat{y}}_{i}$ denotes the true value.

### 3.4. Multi-Objective Optimization Method

At present, the NSGA-II algorithm is suitable for solving nonlinear problems, and, by virtue of its running speed, sound convergence performance, and other advantages [40], it is used for the optimization of mechanical properties of epoxy-bonded CF/QF-BMI composite joints. Figure 3 illustrates the detailed computational workflow that combines the NSGA-II algorithm with the RBF neural network algorithm. To ensure the diversity of the initial population, 200 individuals are randomly generated within the specified range. Then, these 200 individuals are input into the trained RBF neural network model to obtain the tensile and shear strength values as initial fitness values. Next, fast non-dominated sorting is conducted to obtain the fitness level of individuals in the population, and all individuals in front 1 are not dominated by any other solution as the current optimal solution. Front 2 is solely dominated by front 1. According to this principle, continuous layering is carried out to achieve the layer sorting of the target fitness, laying the foundation for the subsequent binary tournament selection. Next, the calculation of individual crowding degree is performed with the following formula:

(23) $$d_{crowding}=\sum_{m=1}^{M}\frac{f_{m}(i+1)-f_{m}(i-1)}{f_{m}^{max}-f_{m}^{min}}$$

where $d_{crowding}$ is the crowding distance. $f_{m}(i+1)$ and $f_{m}(i-1)$ denote the adjacent solution values of solution i for target m, and $f_{m}^{max}$ and $f_{m}^{min}$ represent the maximum and minimum solutions for target m, respectively. The crowding degree of solutions at the boundary is set to infinity so as to ensure their preservation. Binary tournament selection is used for the screening of individuals, and it prioritizes individuals from superior hierarchical tiers. When all the individuals in a layer cannot be screened, priority is given to the individuals with a larger crowding distance to maintain diversity. A crossover operation is performed on the selected individual $Q_{t1}$ as the parents. Its expression is as follows:

(24) $$\begin{array}{c} c_{1}=0.5\left[(1+\beta )p_{1}+(1-\beta )p_{2}\right]\\ c_{2}=0.5\left[(1-\beta )p_{1}+(1+\beta )p_{2}\right]\\ \beta =\left\{\begin{array}{cccc} (2u{)}^{\frac{1}{\eta_{c}+1}}, & if\;u\leq 0.5 & & \\ {\left(\frac{1}{2\left(1-u\right)}\right)}^{\frac{1}{\eta_{c}+1}}, & otherwise & & \end{array}\right. \end{array}$$

where parents $p_{1}$ and $p_{2}$ generate offspring $c_{1}$ and $c_{2}$, with β being controlled by the distribution parameter $\eta_{c}$. $\eta_{c}$ serves as the crossover distribution index, which is utilized to regulate the degree of similarity between the offspring and parent. u denotes a random number whose range is [0 1].

Crossover enables the combination of superior genes from the parents, guaranteeing the offspring’s excellence in fitness. Next, the mutation operation with the following formula is carried out:

(25) $$\begin{array}{c} x^{\prime }=x+\delta \cdot (x^{U}-x^{L})\\ \delta =\left\{\begin{array}{cccc} (2r{)}^{1/(\eta_{m}+1)}-1, & if\;r<0.5 & & \\ 1-(2(1-r){)}^{1/(\eta_{m}+1)}, & otherwise & & \end{array}\right. \end{array}$$

where r is a random number uniformly distributed between 0 and 1. $x^{U}$ and $x^{L}$ represent the maximum and minimum values of the input variables. $\eta_{m}$, serving as the mutation distribution index, adjusts the magnitude of perturbation to vary the probability of mutation.

Variation introduces minor perturbations to enhance the global search capability of the samples. Offspring $P_{t1}$ is generated through crossover and variation, and merged with the parents, which achieves the elitism strategy to prevent the deletion of excellent parent genes due to crossover and variation. In addition, non-dominated individuals screened out in the process of constant iteration are added to the Pareto frontier, and this cycle repeats until the target requirements are met. Noteworthily, in multi-objective optimization, conflicting objectives create scenarios where optimizing one objective often degrades another objective. Thus, optimization is sought for a solution set, i.e., the Pareto optimal solution set, which represents the optimal trade-off of problems. All the curves between the valid solutions of the objective function within the feasible design space constitute the feasible Pareto frontier in the range. Meanwhile, the TOPSIS method is employed for the trade-off of 200 non-inferior solutions in the Pareto solution set, thus discovering the optimal solution.

## 4. Results and Discussion

### 4.1. Finite Element Model Verification

**Figure 4:**
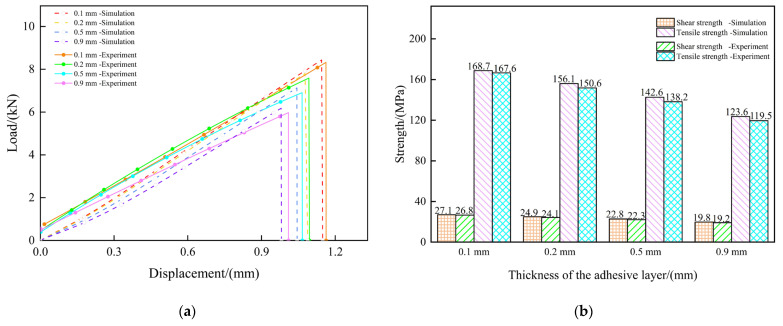
The comparison between experimental results and simulation results: (**a**) the load–displacement curves with different adhesive thicknesses; and (**b**) the tensile and shear strength with different adhesive thicknesses.

The bonding model’s reliability is verified through a comparative analysis of the finite element simulation results and experimental results. The comparative analysis of the bonding performance under varying adhesive film thicknesses is conducted, as shown in Figure 4a. Both experimental and simulated force–displacement curves exhibit that the load first increases with the displacement, and then decreases irreversibly. The error between the simulated and experimental failure loads can be controlled within 3.32%. Although there is still a slight uncertainty in the difference between the experimental data and simulated data, the simulation curve shows the same variation trend as the load–displacement curve obtained in the experiment. This uncertainty in the difference is attributable to deviations between the localized experimental conditions and simplified models, causing the simulation-derived data to be slightly larger than those of the experiment results.

Meanwhile, as observed in Figure 4b, when the thickness of the adhesive film is 0.1 mm, the tensile strength and the shear strength are 167.6 MPa and 26.8 MPa, respectively. As the thickness of the adhesive film increases, the bonding strength shows a downward trend. When the thickness of the adhesive layer reaches 0.9 mm, the tensile strength and the shear strength decrease to 119.5 MPa and 19.2 MPa, respectively. When the length and width of the adhesive layer are fixed, once the thickness of the adhesive layer exceeds 0.2 mm, the mechanical properties of the adhesive layer are significantly weakened. This is mainly because, as the thickness of the adhesive film increases, the shear stress it bears also increases, leading to higher interfacial stress, which makes the structure more prone to failure. The simulation results also reflect this trend, and the simulation–experiment deviation can be explained by the following considerations. Firstly, the inherent irreducible uncertainties in the boundary condition fundamentally constrain the finite element model’s ability to replicate the physical test conditions, particularly at interfacial regions where interactions dominate. The inherent difficulty in maintaining a perfect coaxial alignment between the geometric centroid of the adhesive layer and the loading axis during the testing introduces eccentricity effects. Moreover, both the CF-BMI and QF-BMI composite materials in a single layer are regarded as equivalent homogeneous materials with fiber orientation during modeling. The adhesive layer owns residual stresses during the curing process, while the current modeling approaches adopt simplified stress-free initial conditions. Moreover, it is difficult for the arrangement of carbon fibers and quartz fibers in the samples to meet the ideal arrangement, and it is difficult for the distribution of carbon fibers and quartz fibers in the samples to achieve complete uniformity. In addition, inevitably, there may be some raw manufacturing defects such as voids in the actual epoxy-based film. All of those mentioned above may affect the accuracy of the predicted results.

**Figure 5:**
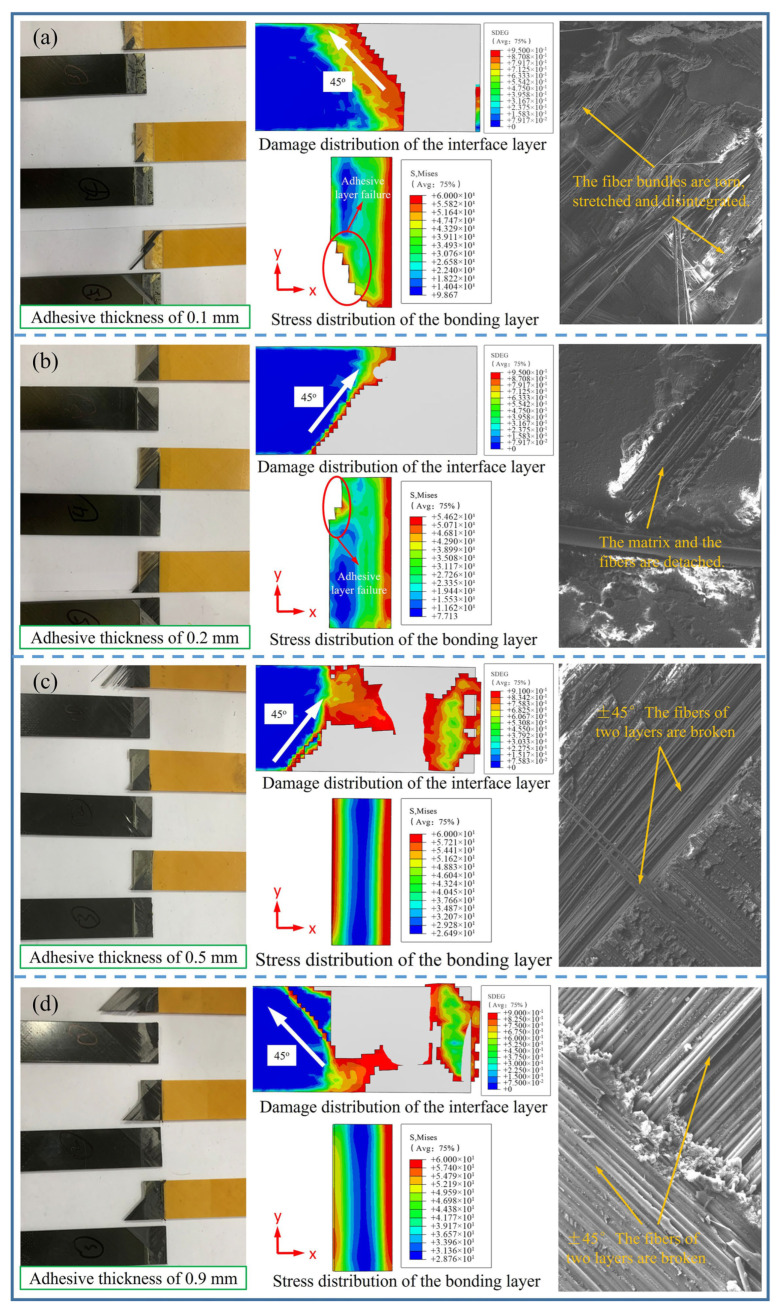
Experimental and simulation results of the component failure modes with different adhesive thicknesses: (**a**) 0.1 mm, (**b**) 0.2 mm, (**c**) 0.5 mm, and (**d**) 0.9 mm.

Both the finite element analysis and simulation cloud chart reveal that the stress distribution of adhesive layers with different adhesive thicknesses is similar, though varying the structural dimensions of adhesive layers lead to distinct failure modes, as shown in Figure 5. Mixed failure modes (adhesive layer failure and ply failure) occur at adhesive thicknesses of 0.1 mm and 0.2 mm. At a 0.1 mm adhesive thickness, the joint witnesses extensive adhesive layer failure. At the same time, small-scale ply delamination and fiber tearing, fracture, and disintegration occur. Among them, ply delamination occurs at a 45° angle along the fiber-laying direction. At a 0.2 mm adhesive thickness, the component exhibits adhesive layer failure, ply delamination, and fiber tearing and fracture. Specifically, ply delamination consistently propagates at a 45° angle along the fiber-laying direction, and the ply delamination-affected area expands compared to that at a 0.1 mm adhesive thickness. When the adhesive thickness increases to 0.5 mm, ply failure spreads from the adhesive layer zone to the adjacent area, and the ply failure area increases. At a 0.9 mm adhesive thickness, the failure is predominantly manifested as ply delamination adjacent to the adhesive layer in the laminate and fiber tearing and fracture (including the fracture of two layers of fiber at ±45°), showing a failure pattern of continuous delamination. Variations in the structural parameters of adhesive layer designs induce distinct failure modes, consequently impacting the service performance of the epoxy-bonded CF/QF-BMI composite joints. Therefore, developing a novel design methodology through the integration of machine-learning and optimization algorithms enables precision improvements in the mechanical properties of epoxy-bonded CF/QF-BMI composite joints.

### 4.2. Machine-Learning Result Analysis

**Figure 6:**
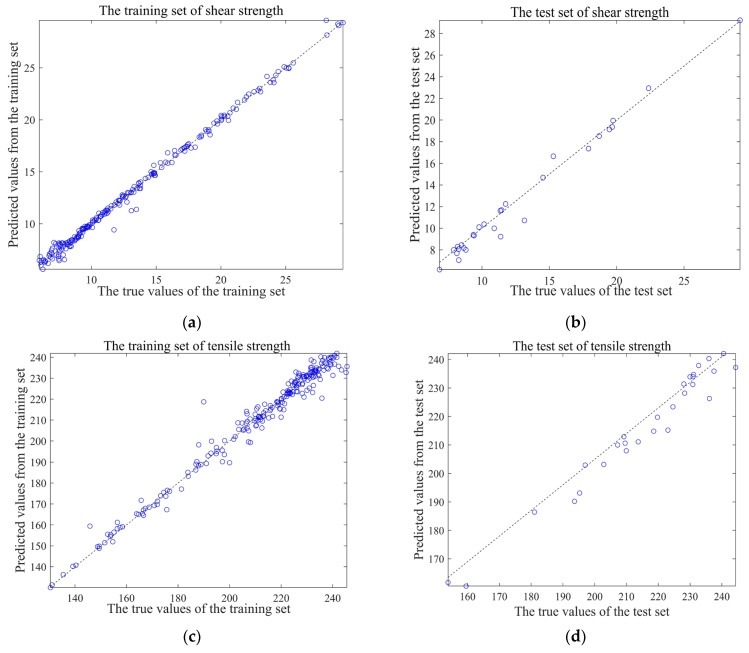
RBF neuron model algorithm fitting performance metrics diagram: (**a**) shear strength training set, (**b**) shear strength testing set, (**c**) tensile strength training set, and (**d**) tensile strength testing set.

A total of 300 different bonding parameters and the tensile strength and shear strength derived from finite element models are input into the RBF neuron model. The RBF neuron model utilizes 260 data as training samples and the remaining 40 data as test samples for machine learning. Then, data normalization and network creation are conducted, including the setting of the RBF neuron model spread speed and the number of neurons in each hidden layer of the feedforward neural network. The detailed model analysis and training results are supplemented, according to Figure 6. The figures in this group represent the evaluation of the accuracy of the trained model in predicting the tensile strength and shear strength. The more data points are on the dotted line in the figures, the better the model fits. As shown in Figure 6a–d, the trained model exhibits outstanding performance across the training and testing sets for tensile strength and shear strength, enabling the implementation of subsequent optimization algorithms. Through the calculation of R^2^, MAE, and MBE, it is concluded that this machine-learning model makes accurate predictions, as shown in Table 3.

**Figure 7:**
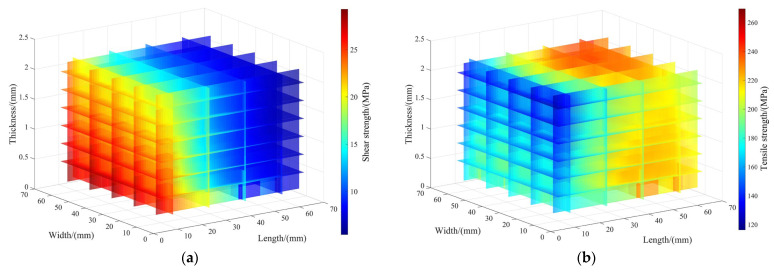
3D slice cloud chart showing the effects of different adhesive joint lengths, thicknesses, and widths on tensile strength and shear strength: (**a**) effects of different adhesive joint lengths, thicknesses, and widths on shear strength; and (**b**) effects of different adhesive joint lengths, thicknesses, and widths on tensile strength.

The trained RBF neuron model can accurately predict the mechanical properties of epoxy-bonded CF/QF-BMI composite joints within the learning range. A 3D slice cloud chart illustrating the effects of different bonding lengths, thicknesses, and widths on the tensile strength and shear strength is shown in Figure 7. At a constant bonding width and adhesive layer thickness, with an increase in the bonding length, the tensile strength of the bonded joint increases rapidly at first, then increases slowly, and, finally, tends to stability, while the shear strength decreases gradually. This behavior occurs because an increased bonding length expands the adhesive bonding zone, while the ultimate failure load tends to stabilize. At a constant bonding length and adhesive layer thickness, both the tensile strength and the shear strength of the bonded joint increase very slowly and show almost no change with an increase in the bonding width due to a linear growth in the ultimate failure load with the increase in the bonded joint width. At a constant bonding width and bonding length, both the tensile strength and the shear strength of the bonded joint gradually decrease with an increase in the adhesive layer thickness because the eccentricity of the epoxy-bonded CF/QF-BMI composite joint grows as the adhesive layer thickness increases. A strong nonlinear relationship between the bonding strength and adhesive layer thickness emerges when the adhesive layer thickness is <0.9 mm and the bonding length is <50 mm, while a weak nonlinear relationship between the bonding strength and adhesive layer thickness occurs when the adhesive layer thickness is >0.9 mm and the bonding length is >50 mm.

### 4.3. Multi-Objective Optimization Results

**Figure 8:**
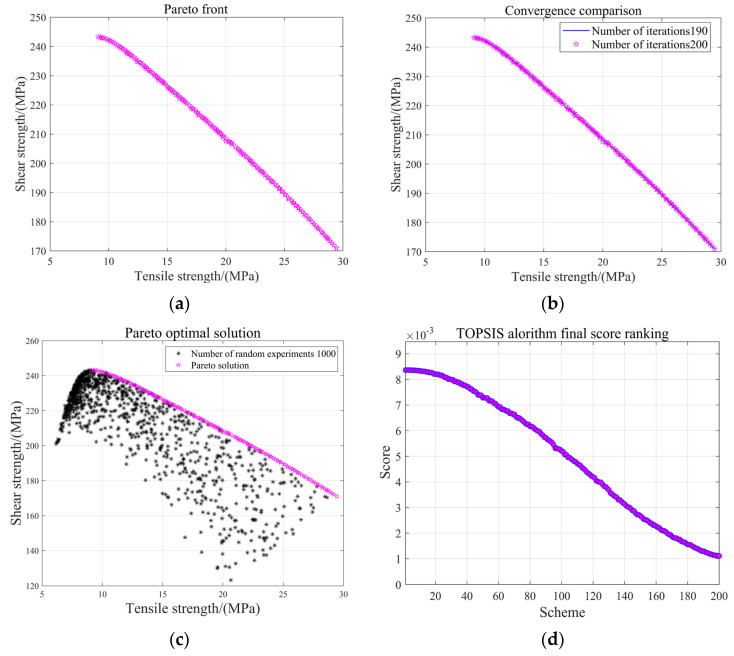
NSGA-II optimization and TOPSIS selection process: (**a**) Pareto front schematic diagram derived from NSGA-II optimization algorithm, (**b**) convergence evaluation schematic diagram, (**c**) Pareto front schematic diagram dominating other solutions within the range, and (**d**) TOPSIS selection process schematic diagram.

The surrogate model for the epoxy-bonded CF/QF-BMI composite joint obtained based on the RBF neural network model optimizes the structural parameters of the adhesive layer by using the NSGA-II algorithm to target the tensile strength and the shear strength. Meanwhile, the initial population size and iteration frequency are set to 200 for the purpose of the effective coverage of the Pareto frontier, thus obtaining global optimal solutions. The crossover probability is set to 0.85 and enhances gene mixing and the global search capability. In order to avoid local optimization in the iteration process, the offspring variation probability is set to 0.007. The Pareto frontiers for the tensile strength and the shear strength within the specified bounds after population evolution are presented in Figure 8.

Figure 8a, in this group of figures, displays the Pareto frontier obtained in the optimization process, including multiple non-inferior solutions. Figure 8b compares the Pareto frontier subject of the 200th iteration with the subject of the 190th iteration. It can be clearly seen that convergence has occurred at the 200th iteration. Figure 8c represents 1000 data points randomly generated in an area based on the Pareto frontier training results, and the observations show that no solution dominates the Pareto frontier, which proves that the optimized Pareto frontier is exactly the optimal frontier in the area. Figure 8d illustrates the score of each non-inferior solution gained according to its weight in the process of TOPSIS optimization. By comparing such scores, we can obtain the optimal parameters of the bonding interface.

The TOPSIS optimization [41] method can be employed for the selection and comparison of the Pareto solution set, in order to find the optimal solution from the non-inferior solutions. Firstly, both the tensile strength and shear strength values of each non-inferior solution are normalized to obtain the normalized decision matrix. Secondly, the normalized matrix is weighted, and the tensile strength and the shear strength employ a weight of 0.5. Then, the positive and negative ideal solutions for each sub-objective are identified from the matrix. Next, the Euclidean distance from the normalized matrix of the corresponding objective function values of each set of combined parameters to the positive and negative ideal solutions is calculated. Finally, the relative approach degree is calculated, and the individual with the maximum relative approach degree is taken as the optimal solution. According to Table 4, the no. 5 bonding parameter combination is the closest to the ideal solution. Therefore, the no. 5 combination can be selected as the optimal parameter solution for epoxy-bonded CF/QF-BMI composite joints. Compared with the conventional solution (L = 12.5 mm, T = 0.2 mm, and W = 25 mm), the final optimization achieved 16.1% and 11.2% improvements in the tensile strength and shear strength of the epoxy-bonded CF/QF-BMI composite joint, respectively. Therefore, compared with the conventional single-parameter single-objective optimization method, the optimal bonding parameter combination obtained using the NSGA-II algorithm is excellent in terms of the multi-objective collaborative optimization capability and solution set comprehensiveness, and it is able to find a set of solutions where multiple objectives are balanced against each other. This demonstrates the superiority of the NSGA-II algorithm in optimizing the structural dimension design of epoxy-bonded CF/QF-BMI composite joints. This method can be used in conjunction with parameter range requirements in actual production experiments to provide a more efficient and reliable design method.

## 5. Conclusions

In this study, based on the experimental results of epoxy-bonded CF/QF-BMI composite joints with different thicknesses, a finite element simulation model considering 3D hashin and cohesion is established. Latin hypercube sampling, coupled with finite element simulation, generates the tensile strength and shear strength data of different adhesive layer structures. The RBF neural network model trained on the simulation data is used to create the surrogate model for the performance of epoxy-bonded CF/QF-BMI composite joints. Finally, NSGA-II algorithm, a multi-objective optimization algorithm, is used to achieve the optimization of multi-objective parameters of the surrogate model within the defined range. The main conclusions of this study are as follows:

(1) The model established based on the 3D hashin criteria and cohesion can accurately predict the failure process of epoxy-bonded CF/QF-BMI composite joints. The stress distribution of adhesive layers is similar under the different structural parameters of the adhesive bonding zone, though varying the structural dimensions of the adhesive layers results in distinct failure modes.

(2) The RBF neuron model’s learning results show that the tensile strength gradually increases with increasing bonding length at a decelerating growth rate, decreases with increasing adhesive layer thickness, and exhibits insignificant variation with increasing bonding width. The shear strength gradually decreases as the bonding length and the adhesive layer thickness increase, while the shear strength shows negligible variation with the increase in bonding width.

(3) The NSGA-II algorithm was used to identify the geometric parameter combination (L = 12.3 mm, T = 0.1 mm, and W = 46.1 mm) that achieved the peak mechanical performance for the adhesive bonding zone of epoxy-bonded CF/QF-BMI composite joints within the parameter range. Compared with the conventional parameter combination for the epoxy-bonded CF/QF-BMI composite joint (L = 12.5 mm, T = 0.2 mm, and W = 25 mm), the tensile strength and the shear strength improved by 16.1% and 11.2%, respectively.

Thus, the combination of RBF machine learning and the NSGA-II algorithm provides a novel methodology for the structural design of the adhesive bonding zone of epoxy-bonded CF/QF-BMI composite joints.

## Figures and Tables

**Table 1:** Mechanical property of the CF-BMI and QF-BMI composites [30,31].

	Materials	Unidirectional CF-BMI Composite Laminate	Woven QF-BMI Composite Laminate
Property			
$\mathrm{Young}’s\;\mathrm{modulus}\;E_{1}$ (MPa)		145,000	30,000
$\mathrm{Young}’s\;\mathrm{modulus}\;E_{2}$ (MPa)		11,000	23,500
$\mathrm{Young}’s\;\mathrm{modulus}\;E_{3}$ (MPa)		11,000	9000
$\mathrm{Poisson}’s\;\mathrm{ratio}\;\nu_{12},\;\nu_{13}$		0.28	0.15
$\mathrm{Poisson}’s\;\mathrm{ratio}\;\nu_{23}$		0.43	0.45
$\mathrm{Shear}\;\mathrm{modulus}\;G_{12},\;G_{13}$ (MPa)		5000	6500
$\mathrm{Shear}\;\mathrm{modulus}\;G_{23}$ (MPa)		4000	5500
$\mathrm{Longitudinal}\;\mathrm{tensile}\;\mathrm{strength}\;X_{t}$ (MPa)		2400	560
$\mathrm{Longitudinal}\;\mathrm{compressive}\;\mathrm{strength}\;X_{c}$ (MPa)		1400	360
$\mathrm{Transverse}\;\mathrm{tensile}\;\mathrm{strength}\;Y_{t}$ (MPa)		100	560
$\mathrm{Transverse}\;\mathrm{compressive}\;\mathrm{strength}\;Y_{c}$ (MPa)		300	360
Shear strength *S* (MPa)		110	70
$\mathrm{Interface}\;\mathrm{Elasticity}\;E_{nn}$$,E_{ss}$$,E_{tt}$ (MPa)		130,000	110,000
$\mathrm{Maximum}\;\mathrm{normal}\;\mathrm{traction}\;t_{n}$ (MPa)		60	53
$\mathrm{Maximum}\;\mathrm{shear}\;\mathrm{traction}\;t_{s},\;t_{t}$ (MPa)		40	32
$\mathrm{Toughness}\;\mathrm{in}\;\mathrm{tension}\;G_{n}$ (kJ/m^2^)		0.58	0.51
$\mathrm{Toughness}\;\mathrm{in}\;\mathrm{shear}\;G_{s},\;G_{t}$ (kJ/m^2^)		1.59	1.55

**Table 2:** Mechanical property of the epoxy adhesive film [32].

	Materials	Mechanical Parameters of Epoxy Adhesive Film J-116
Property		
Young’s modulus *E* (MPa)		2000
Poisson’s ratio *v*		0.3
Yield stress *σ_y_* (MPa)		60
Fracture energy *G_f_* (kJ/m^2^)		0.5

**Table 3:** Performance data for RBF neural network fitting.

	Models	R^2^	MAE	MBE
Strength				
Shears strength		0.99509	0.1611	−0.048091
Tensile strength		0.98788	1.826	0.011954

**Table 4:** Sorting results of multi-objective optimization scheme for epoxy-bonded CF/QF-BMI composite joints.

Sample Number	Parameter Value			Target Value	
	T (mm)	L (mm)	W (mm)	*σ_S_* (MPa)	*σ_Τ_* (MPa)
1	0.1	10	49.1	29.5	173.6
2	0.1	18.6	46.1	22.9	199.7
3	0.1	13.3	56	26.6	185.2
4	0.1	16.7	50.5	24.2	195.1
5	0.1	12.3	46.1	27.7	181.2
6	0.18	36.6	58.1	12.7	232.9
…	…	…	…	…	…
196	0.94	44.5	66	10.3	240.3
197	1.01	49.6	62	9.3	242.5
198	1.04	47.1	67.2	9.7	241.8
199	1.1	48.4	64.1	9.5	242.3
200	1.24	49.6	66	9.3	242.4

## Data Availability

The original contributions presented in this study are included in the article. Further inquiries can be directed to the corresponding authors.
